# Emotional contagion to vocal smile revealed by combined pupil reactivity and motor resonance

**DOI:** 10.1038/s41598-024-74848-w

**Published:** 2024-10-23

**Authors:** Annabelle Merchie, Zoé Ranty, Nadia Aguillon-Hernandez, Jean-Julien Aucouturier, Claire Wardak, Marie Gomot

**Affiliations:** 1 INSERM, Imaging Brain & Neuropsychiatry iBraiN U1253, Université de Tours, Tours, 37032 France; 2 FEMTO-ST Institute, CNRS, Université de Bourgogne Franche Comté, Besançon, France; 3 STMS Lab IRCAM, CNRS, Sorbonne Université, Paris, France

**Keywords:** Motor resonance, Emotional contagion, Pupil reactivity, fEMG, Vocal smile, Cognitive neuroscience, Emotion, Human behaviour

## Abstract

The interplay between the different components of emotional contagion (i.e. emotional state and facial motor resonance), both during implicit and explicit appraisal of emotion, remains controversial. The aims of this study were (i) to distinguish between these components thanks to vocal smile processing and (ii) to assess how they reflect implicit processes and/or an explicit appraisal loop. Emotional contagion to subtle vocal emotions was studied in 25 adults through motor resonance and Autonomic Nervous System (ANS) reactivity. Facial expressions (fEMG: facial electromyography) and pupil dilation were assessed during the processing and judgement of artificially emotionally modified sentences. fEMG revealed that *Zygomaticus major* was reactive to the perceived valence of sounds, whereas the activity of *Corrugator supercilii* reflected explicit judgement. Timing analysis of pupil dilation provided further insight into both the emotional state and the implicit and explicit processing of vocal emotion, showing earlier activity for emotional stimuli than for neutral stimuli, followed by valence-dependent variations and a late judgement-dependent increase in pupil diameter. This innovative combination of different electrophysiological measures shed new light on the debate between central and peripherical views within the framework of emotional contagion.

**Supplementary Information:**

The online version contains supplementary material available at 10.1038/s41598-024-74848-w.

## Introduction

Emotional contagion corresponds to a tendency to infer and react to the sensory, motor, physiological and affective states of others^1^. Three invariable characteristics can be identified: it is primitive, automatic, and implicit. This process enables humans to understand and feel others’ emotions, thoughts and intentions^2^. Emotional contagion encompasses both the emotional state (possibly through automatic mapping of emotions^3^) and its expression in the receiver, such as congruent facial expression through the motor resonance process^4^ However, the role of facial output has been widely debated, as it could constitute both a readout of the emotional state and an input for the subjective experience of emotion. Some authors have even argued in favor of the primacy of facial expression over emotional experience^5^, although the effect of facial feedback on subtle emotion remains a controversial issue^6,7^. The hypothesis of a facial feedback, supports more comprehensive theories according to which the emotional state is modulated by feedback from the peripherical bodily sensations^8^, as opposed to central theories that postulate that they would reflect independent components of the emotional response^9^. A complementary theoretical framework suggests that the emotional state would be largely influenced by cognitive appraisal, which is described within the Component Process Model (CPM) as fast and unconscious^10,11^.

Previous studies have explored how facial feedback both accompanies and potentially influences the perception of emotional stimuli, as well as how it modulates *subjective* emotional experience^12^. Additionally, these studies have examined the role of cognitive appraisal (i.e., any perceptual or cognitive process) in shaping facial expressions, through unconscious mimicry^13^. However, in the framework of emotional contagion, studies have never comprehensively described the interplay between facial motor resonance and *objective* markers of emotional states, whether during implicit or explicit appraisal of emotion. The present study proposes to fill this gap by using objective physiological measures of the two components of the emotional response during the judgement of subtle vocal emotions.

A well-known example of emotional contagion is motor resonance to visual smile. It has already been detected in infants^14^ and in several species^15^. Because smiling is an important social cue, the automatic response to someone else’s smile contributes to the matching the sender’s feeling^16,17^, the synchronization of feelings^18^ and social reciprocity^19^.

Emotions can also be communicated vocally, through modulations of several acoustic cues (pitch, timbre, speech rate) that constitute emotional prosody. Smiling is a great example of mouth position modulation. It involves the bilateral contraction of *Zygomaticus major* (ZM) muscles^20^ that induces mouth retraction, leading to voice-changing vocal tract modifications. Stretching the lips causes a reduction in vocal tract length, and an increase in formants frequency, making the voice brighter^21^. Auditory cues have been shown to be sufficient to identify a smile on the basis of voice alone^22–24^. Vocal smile has a robust mental representation in adults, characterized by an upward shift in the frequency of F1 and F2 formants and an increase in energy in F2, F3 and F4^25^.

Facial motor resonance is thus possible in response to auditory information, as demonstrated by the many studies that have explored reactions to highly salient stimuli such as a baby’s cry or the cheering of a crowd from the IADS^26^ (IADS: International Affective Digitized Sounds) sound database^27–29^. A contraction of ZM in response to pleasant vocalizations and a relaxation in response to unpleasant sounds have also been observed^29^. Hietanen et al.^27^ used vocal expressions of affection (i.e. ‘Sarah’ pronounced with 10 different emotional expressions, such as astonishment and sadness) that resulted in congruent facial motor resonance.

It has been shown that motor resonance to visual emotional stimuli is unconscious^30,31^ and may reflect implicit emotion processing^13^. However, it should be noted that for vocal affect, motor resonance has been elicited only under attentional conditions, while participants were actively evaluating the stimuli they were listening to^31^. This makes it difficult to dissociate implicit and explicit contributions. Arias and colleagues^32,33^ designed prosodic filters based on the perceptual representation of the vocal smile that resulted from previous research involving a psychophysical task^25^. By using this filter to create artificially smiling sentences, they showed a congruent facial motor resonance with an increase of ZM activity in response to smiling sentences and an increase of *Corrugator supercilii* (CS) activity in response to sentences rated as unsmiling in sighted adults^33^ but also in congenitally blind participants^34^. Interestingly, in these studies ZM activity increased for smiling sentences even when participants did not rate them correctly, suggesting that implicit vocal smile processing elicits a motor resonance that would be at least partly independent of explicit judgement. In contrast, CS activity was directly associated with the rating. This indicates that the paradigm used by Arias and colleagues makes it possible to distinguish between implicit processes and explicit appraisal involved in emotional contagion.

In parallel to facial muscle resonance, other physiological indices reflect the emotional state of the receiver. As an objective^35^ and face located index of the Autonomic Nervous System (ANS), pupil reactivity reflects the activation of the locus coeruleus-norepinephrine system^36^ and sympathetic activity^37–41^ but also the inhibition of the parasympathetic Edinger-Westphal nucleus^42^ related to emotional processing.

Prochazkova defined a model of Neurological Mechanisms of Emotional Contagion (NMEC) in which the emotional state of the ‘sender’ is reflected by the ANS of the ‘receiver’ to converge in a common physiological (i.e. pupil dilation and motor contagion) and cognitive state^43^.

Pupil reactivity in response to emotion has been widely studied through the visual modality^37,38,44,45^. Greater pupil dilation has been detected in response to emotional facial expressions than in response to neutral facial expressions. In addition, pupil dilation has been shown to be modulated by the ecological character of the stimulus: the more natural (i.e. dynamic rather than static) the stimulus, the more the pupil dilates^38^. The nature of emotions also has an impact on pupil reactivity. For example, happy faces induce a larger pupil dilation compared to sad faces^46^, or conversely according to other authors^38^. Although inconsistent results have been reported, there is broad consensus on the effect of stimulus valence on pupil dilation. In the visual modality, face-to-face interaction generally leads to a synchronization of pupil size. Whether this synchronization is purely related to pupil mimicry^47^ or reflects an autonomic response to a salient socioemotional stimulus remains unknown. One way to disentangle these two processes is to study pupil reactivity to non-visual stimuli.

Sounds, including emotional sounds such as vocalizations or voices, also induce pupil dilation^48–50^ that is modulated, as is the case with visual stimuli, by authenticity^51^. Strong auditory emotions through vocalizations, whether positive (baby laughing) or negative (a couple arguing), induce pupil dilation^52,53^, with a larger effect for negative than for positive stimuli^39^. In the context of emotional prosody, a difference between negative and positive emotions has been observed, with negative emotional sentences inducing a stronger pupil dilation than positive emotions, in a protocol using very salient emotions from the IADS database^54^, while other studies described the opposite effect. However, the study of pupil reactivity to emotional sounds is still in its infancy and has not yet been extended to ambiguous or subtle emotions.

The main objective of the current study was twofold:

(i) To distinguish between the different components of emotional contagion (i.e. motor resonance and emotional state) and to gain insight into their interplay.
(ii) To assess how these components reflect implicit processes and/or a potential explicit appraisal loop that would reinforce vocal emotional contagion.

For this purpose, we investigated facial motor resonance and autonomic reactivity (pupil dilation) to vocal smile. These indices were combined so that the various components of emotional contagion could be identified. To unravel implicit and explicit motor resonance to vocal smile, facial muscle activity (ZM and CS) was measured while participants listened (processing phase) and rated (judgment phase) standardized sentences containing subtle emotions. Using subtle emotions would help prevent saliency effects and estimate the implicit processing through poorly-recognized trials.

## Results

### Validation of vocal smile model

According to an inverse correlation estimate of the vocal smile model^25^, the smiling effect is characterized by an increase and shift of energy in F1 and F2, and by an increase of energy in F3 and F4. Modulations (referred to as Smiling and Unsmilling Filters) were applied on semantically and prosodically neutral sentences (see Supplementary materials).

To confirm that the participants in the current study used the same acoustic cues to characterize the vocal smile as Ponsot et al., the same reverse correlation task was performed^25^. Results revealed that the vocal smile model was identical in this new group of participants. A more detailed analysis of the reverse correlation results is presented in the Supplementary materials (Figure S1).

### Accuracy

In the present study, artificial prosodic modifications were correctly recognized by participants (Smiling and Unsmiling Filters) with an accuracy of 61 ± 5% (mean ± sd), above 50% (t(24) = 11.55, *p* < .001).

### Motor resonance

Considering that the aim of this work was to compare the muscular activity for Filter, Choice and Response, neutral sentences were removed from the analysis.

Because the two muscles have distinct activity, two separate analyses were performed (see Supplementary materials, Figure S2).

The permutations test revealed significant differences between 3000 and 3400 ms for the Filter condition on *Zygomaticus major* (ZM) activity and between 1500 and 3500 ms for the Choice condition on *Corrugator supercilii* (CS) activity. To standardize the analyses, amplitude measurements were performed over a common 500 ms time window (2900-3400 ms) for each muscle and condition.

#### Zygomaticus major (ZM) activity

**Fig. 1:**
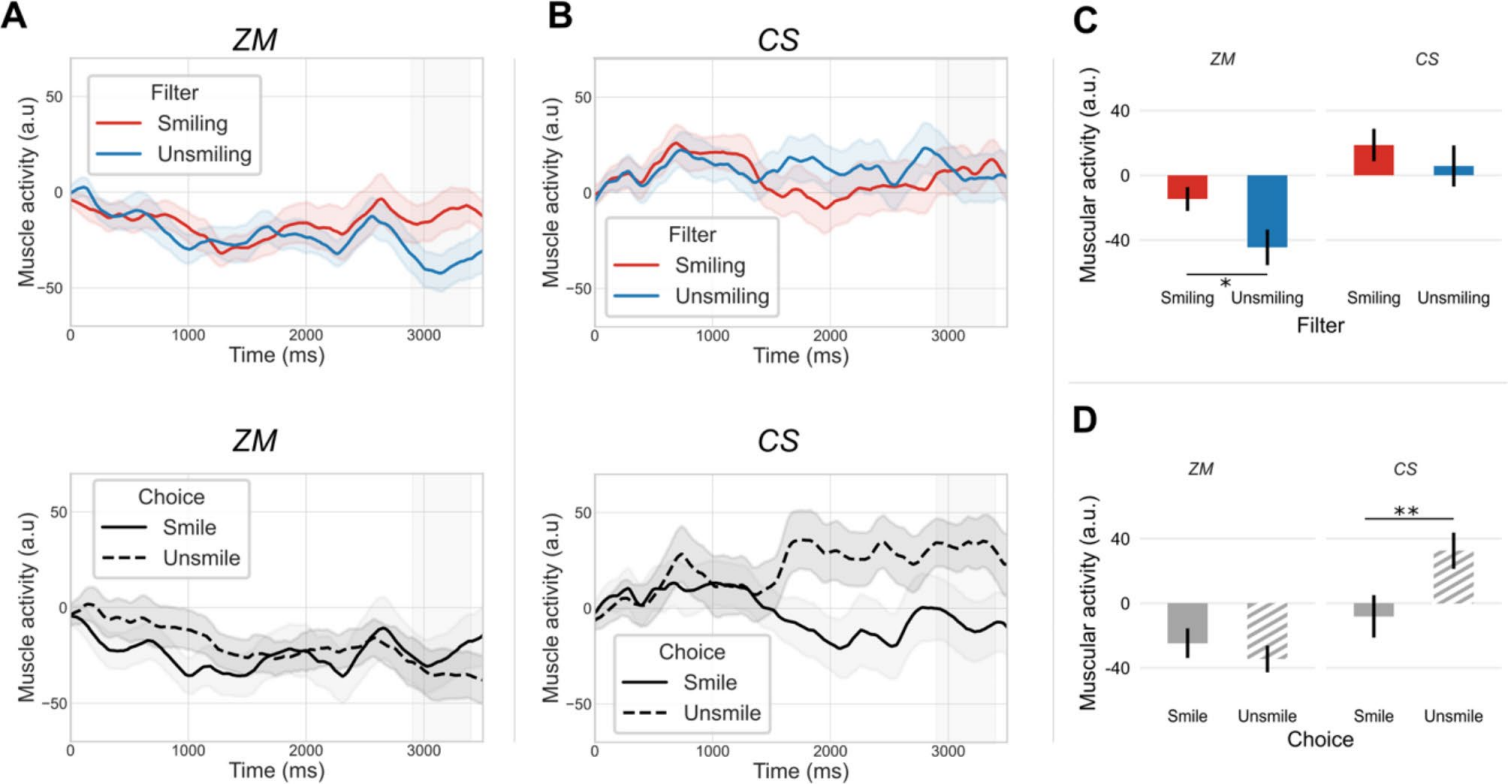
Zygomaticus major (ZM) and Corrugator supercilii (CS) activity. A: Effect of filter (upper panel) and choice (lower panel) on ZM; B: Effect of filter (upper panel) and choice (lower panel) on CS; Effect of filter (C) and of the given choice (D) on CS and ZM -mean response amplitude in the window 2900-3400ms (in grey in A and B time series). Shaded areas represent standard error of the mean. *p < .05; **p < .01.

A repeated-measures ANOVA was performed to explore the effects of Filter (Smiling vs. Unsmiling), Choice (Smile vs. Unsmile) and the interaction between these two factors on mean muscular activity of ZM in the 2900-3400ms time window. The main effect analysis revealed a significant effect of Filter (F(1,23) = 4.73, *p* < .05, η^2^ = 0.06) (Table 1) (Fig. 1A upper part and Fig. 1C). A Smiling sentence induced higher activity of ZM than an Unsmiling sentence (Fig. 1C). Meanwhile, there was no main effect of Choice (F(1,23) = 0.66, *p* > .05, η2 = 0.007) (Table 1) (Fig. 1A lower part and Fig. 1D) and no interaction between Filter and Choice (F(1,23) = 0.28, *p* > .05, η^2^ = 0.002). A repeated-measures ANOVA considering the effects of Choice (Smile vs. Unsmile) and Response (Correct vs. Incorrect) did not yield different results (see Supplementary material, Figure S3).

**Table 1:** Mean muscular activity amplitude in the 2900-3400ms window in response to smiling and unsmiling filtered sentences (a.u : arbitrary unit.) (mean ± standard error of the mean), ZM: Zygomaticus major, CS: Corrugator supercilii.

Condition	Filter		Choice	
	Smiling	Unsmiling	Smile	Unsmile
ZM	-14.7 ± 7	-44.6 ± 11	-24.7 ± 9	-34.5 ± 8
CS	18.7 ± 10	5.8 ± 13	-8.1 ± 13	32.5 ± 11

#### Corrugator supercilii (CS) activity

The same analysis was performed on CS activity with a repeated-measures ANOVA to explore the effects of Filter (Smiling vs. Unsmiling), Choice (Smile vs. Unsmile) and the interaction between these two factors on the mean muscular activity of CS in the 2900-3400ms time window. There was no main effect of Filter on the mean amplitude activity of CS (F(1,23) = 1.88, *p* > .05, η^2^ = 0.01) (Table 1) (Fig. 1B upper part and Fig. 1C) and no interaction between Filter and Choice (F(1,23) = 3.33, *p* > .05, η^2^ = 0.01). In parallel, the main effect analysis showed that Choice (F(1,23) = 10.18, *p* < .01, η^2^ = 0.09) had a significant effect on the mean amplitude activity of CS (Table 1). An Unsmile rated sentence (regardless of the filter) induced higher activity of the CS muscle than a Smile rated sentence (Fig. 1B lower part and Fig. 1D) (Table 1). A repeated-measures ANOVA considering the effects of Choice (Smile vs. Unsmile) and Response (Correct vs. Incorrect) did not show any effect of Response nor an interaction with Choice (see Supplementary material, Figure S4).

### Pupil emotional reactivity

Randomizations tests revealed differences in pupil dilation (PD) between Emotional and Neutral filters from 2100 to 2600 ms. To standardize the analyses, the same time window was used for each condition.

#### Emotional filters

**Fig. 2:**
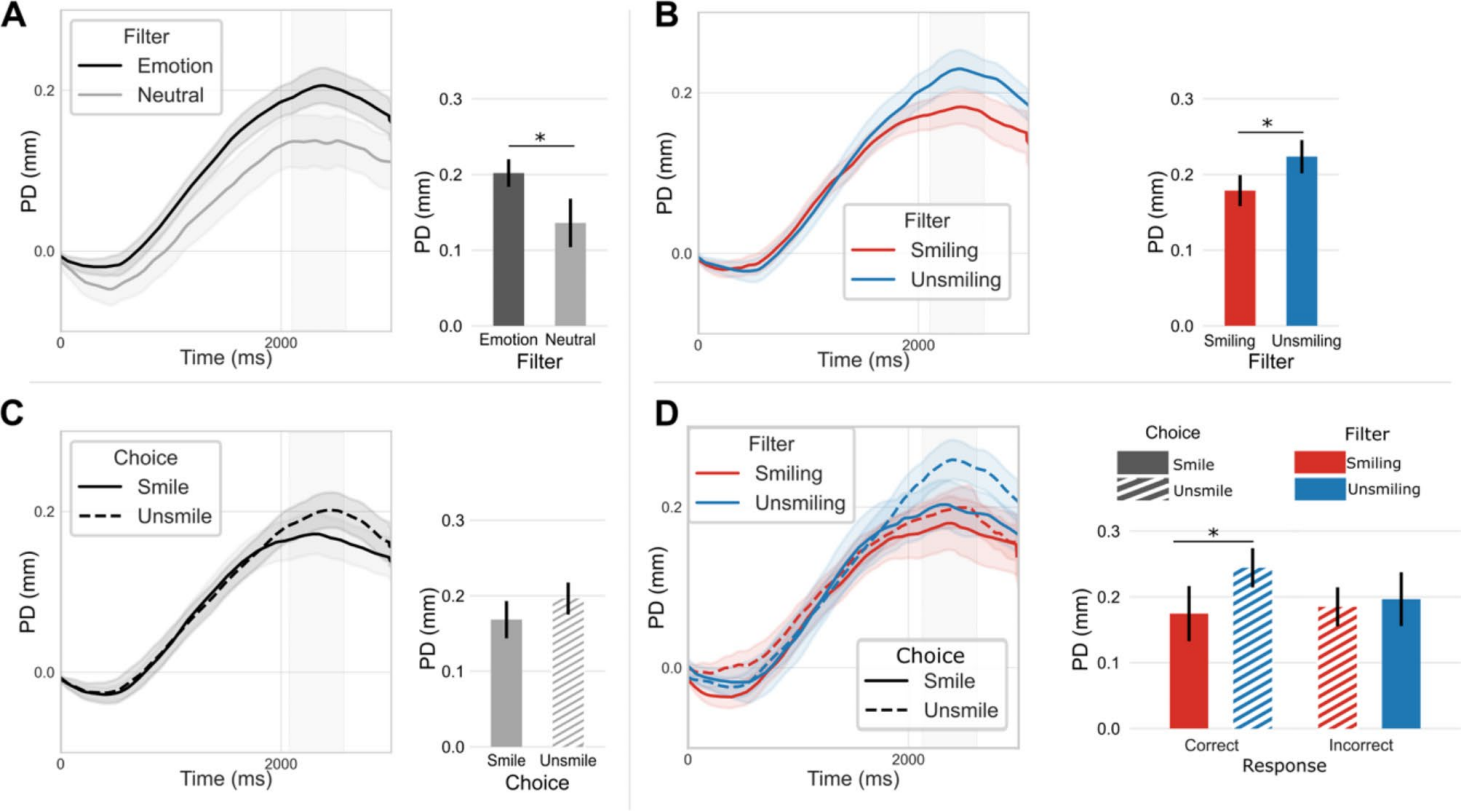
Effect of Filter and Choice on pupil activity. A: Effect of emotional filters on pupil dilation B: Effect of Filter on pupil dilation C: Effect of Choice on pupil dilation (without neutral sentences); D: Effect of Filter, Choice and Response on pupil dilation (neutral sentences excluded). Shaded areas represent standard error of the mean. Barplots represent mean pupil dilation in the window 2100-2600ms (mean ± standard error of the mean) (in grey in time series). PD: Pupil Dilation* *p* < .05.

A repeated-measures ANOVA was performed to analyze the effects of Emotional (both smiling and unsmiling) vs. Neutral filters on the mean PD in the 2100-2600ms window. Emotional filters had a significant main effect (F(1,24) = 6.61, *p* < .05, η^2^ = 0.06) on the mean pupil dilation. Dilation was larger when listening to emotional sentences compared to neutral sentences (Table 2) (Fig. 2A).

**Table 2:** Mean pupil dilation in the 2100-2600ms window in response to neutral and emotional sentences, according to the applied filter, choice and response (mm) (mean ± standard error of the mean).

	Filter			Choice			
	Neutral	Emotional		Smile		Unsmile	
Pupil dilation	0.14 ± 0.03	0.21 ± 0.02		0.17 ± 0.02		0.20 ± 0.02	

		Filter		Filter response		Filter response	
	/	Smiling	Unsmiling	Smiling Correct	Unsmiling Incorrect	Smiling incorrect	Unsmiling correct
Pupil dilation	/	0.18 ± 0.02	0.22 ± 0.02	0.17 ± 0.03	0.20 ± 0.03	0.19 ± 0.02	0.25 ± 0.02

#### Filter and choice

A repeated-measures ANOVA was performed to analyze the effects of Filter (Smiling vs. Unsmiling), Choice (Smile vs. Unsmile) and the interaction between these two factors on mean pupil dilation (PD) in the same time window (2100-2600ms). The main effect analysis showed that Filter had a significant effect (F(1,24) = 4.30, *p* < .05, η^2^ = 0.02) on mean PD, Smiling sentences induced smaller PD than Unsmiling sentences (Fig. 2B) (Table 2).

No main effect of Choice (F(1,24) = 1.93, *p* > .05, η^2^ = 0.02) was observed (Table 2) (Fig. 2C) and there was no significant interaction between Filter and Choice (F(1,24) = 0.48, *p* > .05, η^2^ = 0.004) on mean pupil dilation (Fig. 2D) (Table 2).

A repeated-measures ANOVA was performed to analyze the effects of Choice (Smile vs. Unsmile), Response (Correct vs. Incorrect) and the interaction between these two factors on mean PD in the same time window (2100-2600ms). No main effects were observed either for the Response (F(1,24) = 0.48, *p* > .1, η^2^ = 0.004) or Choice (F(1,24) = 1.93, *p* > .1, η^2^ = 0.02). However, a significant interaction between these two factors was detected (F(1,24) = 4.30, *p* < .01, η^2^ = 0.02). In order to inspect relevant effects, only comparisons within Correct and Incorrect responses were performed with a Bonferroni correction. This analysis revealed a significant difference between Correct responses as a function of Choice (t(24) = 2.45, p_corr_< 0.05) (Fig. 2D). No difference was observed between Incorrect responses (t(24) = 0.097, p_corr_> 0.05).

#### Slopes analysis

**Fig. 3:**
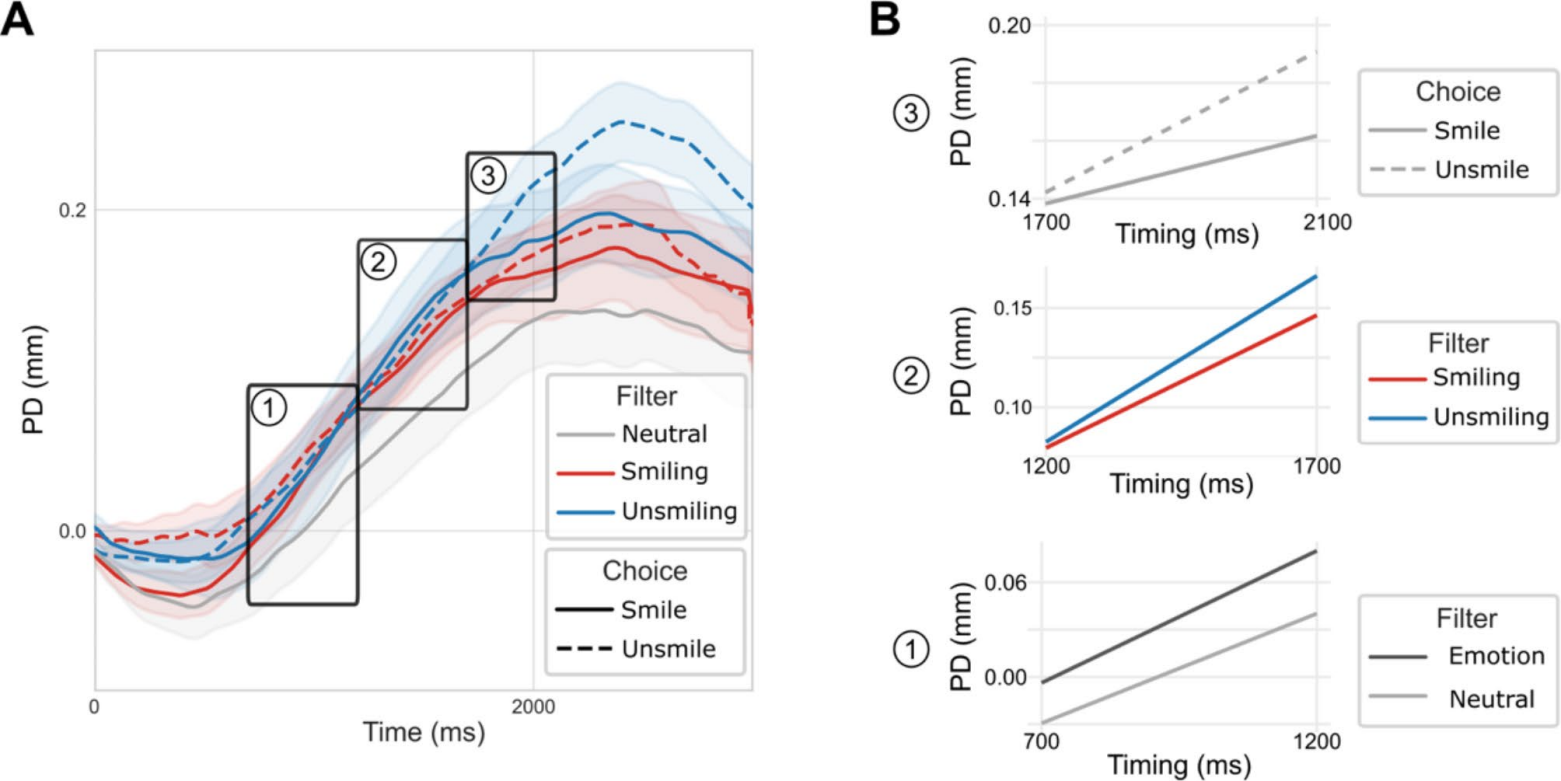
Effect of Filter and Choice on pupil dilation kinetic. A: Effect of Filter and Choice on pupil dilation with Neutral sentences (no matter the judgement for them) with time windows chosen for slopes analysis. B: 1- Effect of emotional content; 2- Effect of Filter; 3- Effect of Choice. PD: Pupil Dilation.

Besides analyzing the mean amplitude, a slope analysis was conducted to assess the kinetics of pupil dilation in response to Emotion, Filter, and Choice across different timing conditions Fig. 3A). A difference was observed between Emotional (combined Smiling and Unsmiling) and Neutral sound in the 700-1200ms time window (β = -2.8e^−5^ with Emotional filter as the reference level; SE = 1.3e^−6^ ; *p* < .001), with the pupil dilation slope being sharper for Emotional than for Neutral sounds (Fig. 3B-1). Note that in this early time window no difference between the slope of Smiling and Unsmiling Filter was revealed. In the next time window (1200-1700ms) a difference in slope was shown as a function of Filter (β = 3.4e^−5^ with Smiling filter as the reference level ; SE = 6.8e^−6^ ; *p* < .0001), with sharper pupil dilation for Unsmiling sounds than Smiling sounds (Fig. 3B-2). Finally, in the last time window (1700-2100ms), an effect of Choice on pupil dilation slope was observed (β = 6.3e^−5^ with Smile choice as the reference level; SE = 2.0e^−6^ ; *p* < .0001), with an increasing curve for Unsmile but not for Smile rated sounds (Fig. 3B-3).

## Discussion

To our knowledge, this study was the first to combine, in the same participants, facial EMG (fEMG) and pupillometry in response to vocal smile. The combination of these physiological measures provided a picture of automatic motor resonance/reactivity at different processing stages through different systems (ANS and facial muscular activity) in response to artificially prosodically modified sentences. The combined analysis of these indices revealed that the emotional state is triggered early and thus, is independent of facial motor contagion. This is evidenced by the fact that autonomic reactivity was sharper to emotional stimuli than to neutral stimuli and preceded facial muscular reactivity. Our paradigm also allowed us to examine more rigorously the facilitating influence of explicit judgement on both muscular and autonomic nervous system activity, suggesting an appraisal and a reinforcement of the already implicitly processed and automatically expressed emotion. Upcoming studies should also investigate these processes to ensure the results repeatability considering our small, though ‘reasonable’ sample size.

Indeed, as in Arias et al. (2018), our results on facial muscular activity made it possible to distinguish between implicit and explicit motor resonance to vocal smile. The *Zygomaticus major* (ZM) reacted to smiling even in the absence of congruent choice, indicating only implicit integration of prosody, whereas *Corrugator supercilii* (CS) activity reflected explicit judgement. The positive correlation between different muscular activities suggests that perception and identification of vocal smile induced congruent implicit and explicit motor resonance. However, even if CS activity reflects the judgement of vocal emotion, no correlation was observed with either accuracy or correct/incorrect responses: correct recognition of emotional prosody was not related to a larger explicit motor resonance.

Consistently, it has been shown that even if expressing facial emotion can help to categorize other’s facial^55^ and vocal emotions^56^, it is not mandatory to recognize emotions. Patients with Moebius syndrome, characterized by congenital facial paralysis, performed similarly to a control group in a facial expression recognition task^57–59^.

Non-social sounds have been shown to induce congruent emotional responses, such as the contraction of ZM muscle in response to a melody rated as ‘happy’ and the contraction of CS muscle in response to a melody rated as ‘sad’^,60^. In the present study, the congruent activity of ZM muscle in response to vocal smile, even when not identified as such (as in Arias et al. studies^33,34^), suggests an automatic emotional facial resonance distinct from music-induced emotional response alone.

The analysis of autonomic reactivity allowed further investigation of emotional contagion. Pupil automatic reactivity would reflect the modulation of emotional state following the perception of a vocal smile. First, pupil reactivity was observed for both emotional and neutral sentences. This systematic dilation reflected low-level sensory processing, cognitive load, and sentence evaluation^61–64^. Second, the artificially modified sentences were sufficiently emotional to induce a larger dilation than unmodified neutral sounds in an early time window, an effect similar to the that observed with natural emotion^46,65^. Third, in a later time window, sound valence influenced pupil reactivity, leading to a larger dilation for negative sentences than for positive sentences. This result has been widely replicated by studies that have focused on the physiological response to sounds^39,53,62^, but only with very salient emotional stimuli. The stimuli included in previous research are highly attention-grabbing, which might lead to cofounding effects that prevent purely emotional aspects from being explored. Our results extend these observations to more subtle stimuli: the sentences used conveyed subtle emotions allowing for finer recognition of acoustic cues and thus for reproducing an effect which is closer to that observed during a real communicative situation. Altogether, our findings point towards the existence of emotional contagion to subtle vocal smile. In the last time window, pupil dilation continues for recognized Unsmiling sentences, suggesting that explicit appraisal modulates pupil reactivity for negative emotion. This increasingly detailed analysis of the emotional content of sentences reflected in the activity of the pupil attests that the pupil is a valuable window into the emotional integration processes at the ANS level.

A recent study demonstrated that ZM muscle innervation includes 10% of ANS fibers (vs. 3.9% for CS)^66^. Thus, in our study, implicit ANS response to sounds the emotional content could also be reflected in ZM reactivity. These findings are consistent with the model of Neurological Mechanisms of Emotional Contagion (NMEC) proposed by Prochazkova: in response to a visual emotion, a ‘sender’ emotion contagion is observed through the ANS on the ‘receiver’^43^. This model, according to results of the present study, could thus be generalized to auditory emotion.

The present study time course contributes to enriching theories of emotions by providing information on the feedback loop between perception of emotions, facial expressions, and their experience. This emotional loop raises several questions concerning the historic debate between peripheralist^8^ and central theories of emotion^9^. Damasio studied the effect of emotional somatic markers on decision-making^3,67^. Regarding somatic response, emotional motor resonance and pupil reactivity may reinforce each other. This idea is supported by the correlation observed between ZM and CS muscle activities, suggesting a reinforcement of facial muscles. The activation of one of the effectors might thus trigger a congruent response of the whole face. Moreover, although the changes in the pupil cannot be intrinsically perceived, they contribute to global physiological emotional response. Even if there is no feedback from the effect of the smooth muscle opening the iris, this leads to a change in the amount of light and information entering the visual system and processed automatically which contributes in its turn to changes in the emotional state. Note that this proposal should be interpreted with caution, as no correlation has been reported between muscular and pupil reactivity.

The current study offered the rare opportunity to jointly observe emotional motor resonance and emotional state modulation via the use of different psychophysiological measures. To go deeper into studying these two phenomena, questioning participants about their subjective feelings towards the filters that have been applied could provide further information on emotional contagion. Within the framework of the polyvagal theory^68,69^, the addition of other ANS measures such as heart rate would provide information about the emotion valence of smiling voices, especially their prosocial valence. Finally, to confirm that implicit effects were the consequence of prosodic modulations rather than artificial characteristics, natural smiling and unsmiling sentences could be added.

## Conclusion

This paradigm used in the present study allowed us to control the intensity and acoustic characteristics of emotional prosody content and thus describe emotional contagion through two different physiological indices.

Emotional contagion is the last process of the perception – representation – action loop^70^. Congruent response to visual smile associated with mimicry in the visual domain. This study disentangles emotional states and motor resonance, together with implicit and explicit aspects of emotional contagion through the use of auditory emotional stimuli and the combination of autonomic and facial motor responses. Mimicry of emotions like laughing and smiling is known to be altered in individuals with disorders such as autism^71,72^. The present paradigm, which includes measures of motor and autonomic reactivity to emotional vocal sounds, could be used to investigate clinical populations. In particular, the artificial modification of sounds through the application of a smiling filter could be pushed one step further: sounds could be artificially modified on the basis of individual models of vocal smile. This would allow measurement of emotional contagion to one’s own vocal smile, opening the way to the investigation of the individual variability that characterizes many disorders.

## Methods

### Population

Twenty-five young adults aged 20 to 30 years (mean 24.0 ± 2.5 years) participated in the study (12 females); all were native French speakers. This sample size was chosen according to other fEMG (Vrana et al., *n* = 19, Rymarczyk et al., *n* = 30 and Sato et al., *n* = 29)^73–75^ and pupillometry (Bufo et al., *n* = 30, Legris et al., *n* = 17, Ricou et al., *n* = 30)^48,76,77^studies’ sample size. An *a posteriori* power analysis was performed (see supplementary materials). To be included in this study, participants should not have (history of) neurological, psychiatric and metabolic disorder, nor be under medication at the time of the study. Each participant completed the Empathy Quotient questionnaire (EQ)^78^, which provides an estimate of empathic abilities.

#### Ethics statement

The study was approved by *Comité de Protection des Personnes* CPP Est I (PROSCEA2017/23; ID RCB: 2017-A00756-47). All methods in this study were carried out in accordance with the relevant guidelines and regulations, and all data in this study were obtained with informed consent from all subjects and/or their legal guardian(s).

### Stimuli and experimental design

#### Stimuli and sequence of stimulation

The same corpus as in Arias et al.’s studies^33,34^ was used (see Supplementary materials). Only semantically neutral sentences were selected, so as to make sure to test the effect of emotional prosody content^79^. The emotional prosody of the sentences was modified via an algorithm including a Smiling and an Unsmiling filter, created following the model of vocal smile proposed by Ponsot et al.^25^. Validating the vocal smile model in this sample was a prerequisite for the validity of modified emotional sentences.

#### Procedure

**Fig. 4:**
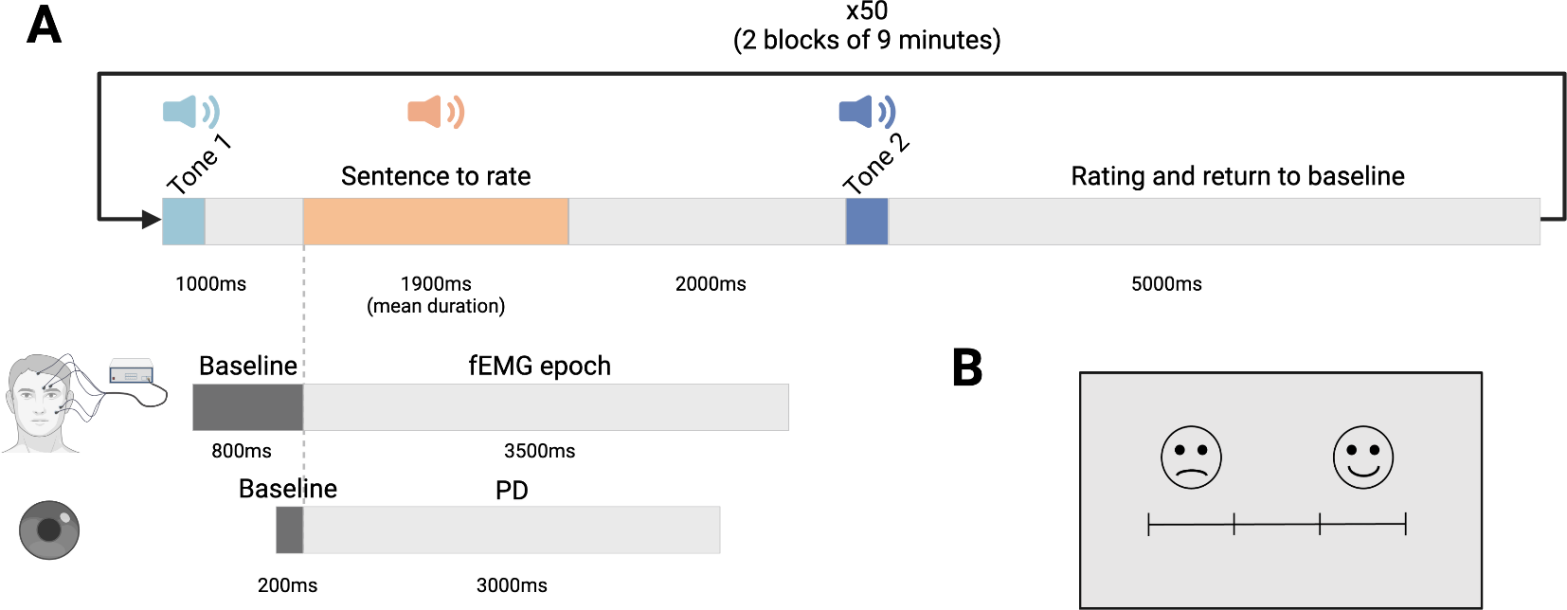
A: Timeline of a trial in the experimental sequence. Tone 1 indicated the beginning of a new trial, tone 2 indicated the time when the subject should rate the sentence. B: Rating scale displayed on screen throughout the duration of the task; PD: Pupil Dilation; fEMG : facial ElectroMyoGraphy.

Two 300 ms tones, each with a specific frequency, were created using Praat^80^ and normalized in energy and faded on in- and output. One tone (440 Hz) was used to signal the beginning of a new trial. The sentence was played 1000 ms later, followed by a silent period of 2000 ms, and finally by the second tone (275 Hz), which indicated the rating time and the return to baseline (lasting 5000 ms). The time course of each trial is represented in Fig. 4A. Because the research question was focused on sound processing, the image of a rating scale displayed on the screen (Fig. 4B) remained unchanged throughout the experiment; successive stages were indicated with tones. Using the same image for the entire time of the experiment allowed us to maintain a constant luminance (around 20 lx), which was a necessary condition to ensure that the observed effects on pupil dilation were a consequence of the auditory task.

The participants were comfortably sitting 70 cm away from the screen (1980 × 1080 px) and the speakers. No chinrest was used. The intensity of the sounds was controlled and maintained between 58- and 62-dB SPL.

The following instruction was given to participants: ‘You will hear sentences, and you will be asked to rate how joyfully or unjoyfully the sentence was pronounced. You should not judge the content of the sentence but the way it was pronounced’. For the behavioral data associated to facial EMG and pupil recording, judgements were recorded through a 4-key response pad. The displayed image represented the rating scale: the two right keys corresponded to two levels of Smile intensity and the two left keys corresponded to two levels of Unsmile intensity.

### Measurements

#### Facial EMG (fEMG) - recording

Facial EMG (fEMG) was recorded with 4-mm Ag-AgCl electrodes EL254 and EL254S, the BIOPAC MP36 acquisition system (BIOPAC^®^ Systems Inc. Goleta, CA, biopac.com) and AcqKnowledge^®^ 4.1 software (biopac.com). In order to measure the activity of the two facial muscles *Zygomaticus major* (ZM) and *Corrugator supercilii*(CS) and following Fridlund and Cacioppo’s guidelines^81^, the electrodes were placed on the left side of the participant’s face after scrubbing with an abrasive paste to remove traces of moisturizer, makeup or perspiration. Data were recorded with a sampling rate of 1024 Hz. For each muscle, two electrodes separated by approximately 1 cm were used. This allowed us to measure the potential difference between the anode and cathode, placed in the same position on each participant’s face. A reference electrode was added to the center of the forehead at the hairline.

#### Pupillometry - recording

Pupil data were recorded using a SMI RED 500^®^ system, synchronized with BIOPAC MP36^®^, at a 500 Hz sampling rate using an infrared light (λ = 870 nm) to detect and measure pupil dilation (PD) in millimeters (mm)^82^. Data were recorded only after a correct 5-point calibration with a deviation degree of less than 1° (mean: 0.59°/0.58°).

### Data analysis

#### Behavioral data analysis

For all analysis, the acoustical modification of the sentences is referred to as Filter (Smiling vs. Unsmiling) and the rating of the degree of smiling in the sentences is referred to as Choice (Smile vs. Unsmile). The congruence between Filter and Choice is referred as the Response, which can be Correct (Smiling rated as Smile and Unsmiling rated as Unsmile) or Incorrect (Smiling rated as Unsmile and Unsmiling rated as Smile).

Accuracy was calculated as the number of Correct responses divided by the sum of all Smiling and Unsmiling sentences (80 for each participant), to reflect the good rating of both filters (Eq. 1). Accuracy was compared to chance level (50%) to evaluate the recognition of emotional prosodic modulation.

1 $$\:Accuracy=\frac{nSmilin{g}_{smile}+nUnsmilin{g}_{unsmile}}{nSmiling+nUnsmiling}$$

Equation FormulaeAccuracy formula; nSmiling_*smile*_: number of trials for smiling sentences rated as smile; nUnsmiling_*unsmile*_: *number of trials for Unsmiling sentences rated as unsmile; nSmiling: total of trials for the Smiling Filter ; nUnsmiling: total of trials for the Unsmiling Filter.*

#### fEMG – processing

Analysis and pre-processing of fEMG data were performed with MNE-toolbox on Python 3.9.4^83^. Data were filtered with a 50 Hz IIR high-pass filter and a 250 Hz low-pass filter. Subsequently, significant muscular activity artifacts were manually removed after visual inspection of the recordings. Data were then segmented into epochs, with an 800 ms pre-stimulus baseline and a duration of 3500 ms, starting from the beginning of each sentence. The absolute value of the muscular activity was smoothed and averaged using a 300 ms sliding window, followed by z-score normalization based on the baseline of the current trial. On average, 20 ± 11% (mean ± sd) of trials per participant were removed due to excessive motion artifacts. One participant was excluded from the fEMG group analysis because of excessive noise in the signal, but data from this participant were retained for pupil and behavioral analysis.

#### Pupillometry – processing

Pre-processing and processing of raw pupil data were performed using in-house MATLAB^®^ (R2015b; MathWorks) scripts. The first step involved detecting and interpolating artifacts caused by blinks and brief signal losses. Blink detection was achieved using a velocity-based algorithm^83,84^ supplemented by manual detection. Interpolation for missing values was performed with a cubic interpolation with the median value. For each trial, a 200 ms baseline before the beginning of a sentence playing was extracted. Pupil Dilation (PD) was calculated as pupil diameter increase in mm in response to stimulus presentation and choice relative to the baseline of the trial. Pupillary response was measured during 3000 ms after the start of sentence playback. Each trial was visually inspected, and 34 ± 22% (mean ± sd) of trials per participant were removed due to excessive artifacts.

### Statistical analysis

Statistical analyses were conducted with Rstudio 4.2.2^85,86^ with the packages ggplot2^87^, ez^88^, tidyverse^89^ and dplyr^90^.

For each physiological measurement (i.e., muscle activity and pupil dilation), the effects of different factors (Filter, Choice, Response) were estimated through the mean amplitude of electrophysiological measures within selected time windows. To assess the differences between conditions in a psychophysiologically relevant time window, randomizations (*n* = 10,000) were performed across conditions (Smiling vs. Unsmiling and Smile vs. Unsmile)^91^ with a Guthrie-Buchwald correction^92^. For the fEMG data randomizations, a down-sampling to 512 Hz was applied to the signal using the MNE-toolbox^93^.

Within the randomizations, a common 500 ms window was selected, in the significant time period, in which average amplitude was measured for different conditions in each physiological measure. To identify the significant effect in the selected time window, a repeated-measures ANOVA (Filter x Choice) was performed on mean muscular activity or PD amplitude. To assess the effect of Response and interaction with Choice, a repeated-measures ANOVA of the mean amplitude of muscle activity and PD was performed with post-hoc pairwise comparisons with Bonferroni correction to further specify significant interaction effects within Correct and Incorrect Response.

In addition to the mean amplitude analysis, an analysis of slopes was performed in order to evaluate the kinetics of pupil dilation in response to Emotion, Filter and Choice as a function of Timing. A linear mixed model was performed with Emotion, Filter and/or Choice and Timing as fixed factors and with subjects as random factor and Emotion, Filter and/or Choice as random slopes with the lmer R library^94^. This analysis was performed by taking into account different time windows after signal visual inspection: 700 to 1200 ms for Emotion, 1200 to 1700 ms for Filter and 1700 to 2100 ms for Choice effect.

Finally, a Pearson correlation matrix was computed between physiological measures, rating responses, and EQ scores to explore the relationships among all measures. A Bonferroni correction was applied for multiple comparisons. Results are presented in Supplementary materials (Figure S5).

## Electronic supplementary material

Below is the link to the electronic supplementary material.

Supplementary Material 1

## Data Availability

Code and data for processing and statistical analysis are publicly available in an OSF project (https://osf.io/6rus4/).
