# Supplementary material for: Emotional contagion to vocal smile revealed by combined pupil reactivity and motor resonance
Source: Sci Rep. 2024 Oct 23;14:25043. doi: 10.1038/s41598-024-74848-w (PMC11499673; doi:10.1038/s41598-024-74848-w)
Supplement: Supplementary file 1 — Supplementary Material 1 [file 41598_2024_74848_MOESM1_ESM.docx]

**Supplementary materials**

# Corpus of sentences used

The corpus consisted of five different sentences, each pronounced by ten different speakers, with neutral semantic content designed to evaluate the effect of prosody [1]. A total of 100 sentences, with an average duration of 1900 ms, made up the sequence. Smiling and unsmiling sentences were repeated twice as often as their neutral counterparts, resulting in 20 neutral, 40 unsmiling, and 40 smiling sentences. The sequence of sentences was arranged to ensure that there were never:

- Two consecutive sentences pronounced by the same speaker,
- Two consecutive sentences with the same linguistic content,
- Three consecutive sentences with the same filter/prosody,
- Three consecutive sentences pronounced by speakers of the same gender.

The unmodified neutral sentences were included in the sequence to balance the filters and reduce habituation to the stimulus.

# Sample size estimation

This study was the first combined exploratory study on pupil and facial reactivity to subtle emotional prosodic cues. For this reason, no previous similar study reporting size effect was available, preventing us from estimating an appropriate sample size prior to our study. Nonetheless, the number of trials for each participant was large (n=80). To justify the sample size, we conducted an *a posteriori* power analysis based on Arias et al., 2018 [2] reported size effect on muscular activity (with only 60 trials per subject and a sample of 35 participants) on our 25 participants (power = 0.58 for ZM and power = 0.38 for CS). Despite this limitation, this study provides for the first-time insights on emotional contagion and further work should be done to enrich literature on this phenomenon in response to vocal emotion.

# Validation of vocal smile model

To validate that the vocal smile model used to artificially modify sentences in this study was the same as in Arias et al.'s [2,3] work, the same task as in Ponsot et al. [4] was performed. However, to enhance feasibility, the task duration was reduced significantly by decreasing the number of trials to 300 (compared to 600 trials in the original task). The task was divided into six blocks of 50 trials each. The last two blocks were identical to measure the internal consistency of the participants' model, estimate its robustness using the double-pass methodology, and assess internal noise [5,6,7].

The methodology was the same as in Ponsot et al. [4]. This task was based on reverse correlation analysis: in each trial, participants compared two different sounds created from the same original sound and chose the one that sounded the most "smiling." The sounds were modified using the open-source CLEESE toolbox [8] from a 500 ms sound the vowel /a/ pronounced by a male speaker. For a detailed description of the sound modifications and analysis procedures, see Ponsot et al. [4]. To compare the two models of vocal smiles, a statistical comparison of spectral modifications was performed on each modified frequency.

The data of two subjects were removed due to excessive deviation from the control model of Ponsot et al. [4].


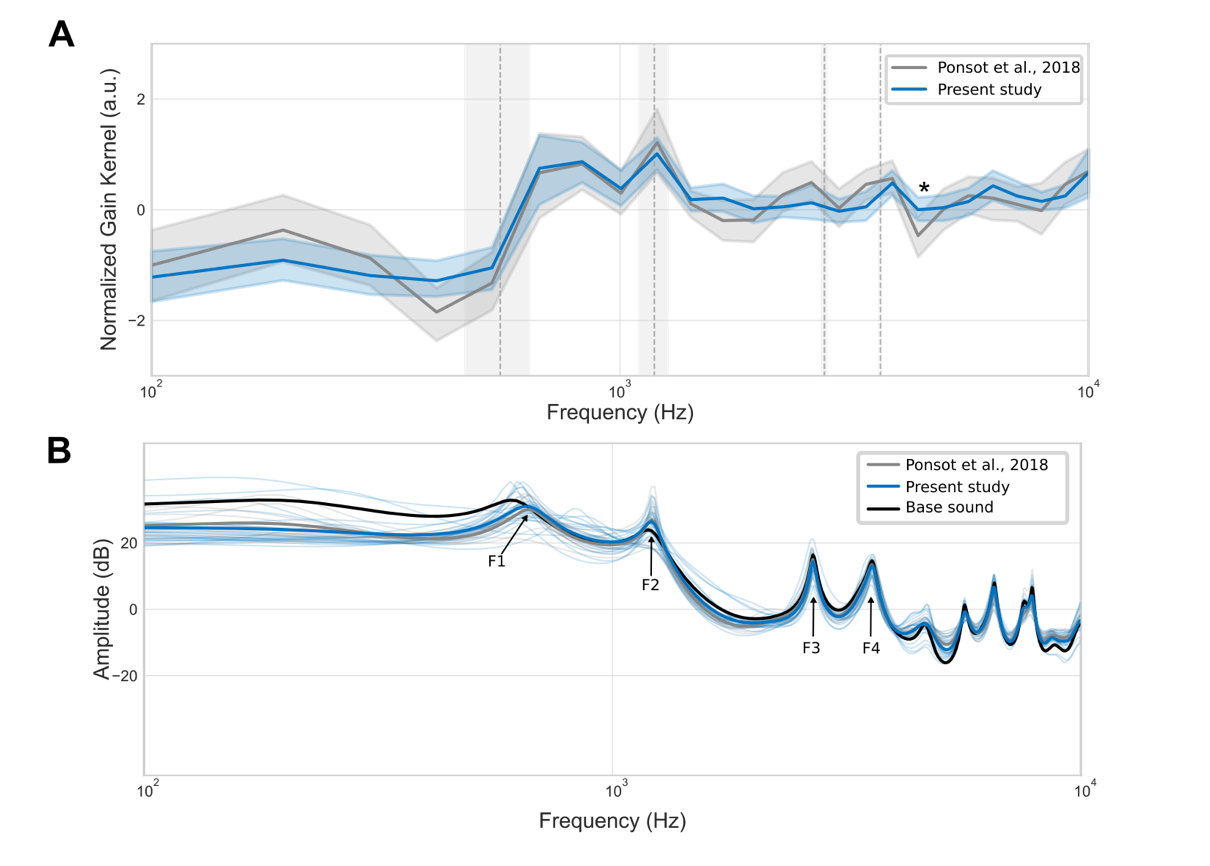


Figure S1: A: Averaged filter underlying the judgment of vocal smile, as derived with reverse-correlation. Asterisks indicate significance differences between the two groups (two-tailed; paired-sample t-tests, p<.05). Vertical shaded areas indicate how the first four formants of the voice align with the structure of the Filter. B: Smiling Filter is applied (here with a gain of 0.5) to the original voice, it reveals the internal representation of vocal smile in the two groups compared to the base sound.

The obtained smiling kernel was applied to the original sound and spectral envelope was extracted and compared between groups (Figure S1). An analysis of the acoustic features was performed to determine if formants are modified by the smiling effect. o study the modifications of frequencies after the application of the smiling filter, the first four formants were extracted using Praat [9]. The results are reported in Table S1.

To determine the influence of vocal smile modifications on the original sound, a comparison analysis was performed between the formant values of the original sound and after modification using a one-sample t-test. The results, along with a comparison between the results of Ponsot et al. [4] and the present study, are reported in Table S1. The only frequency for which there was a significant difference between the two groups was 4332 Hz (p < .05). To conclude, as in Ponsot et al., the current sample of participants, even with a smaller number of trials, demonstrated that the mental representation of a vocal smile was the same.

| **Original sound** | **F1** (555 Hz) | **F2** (1183 Hz) | **F3** (2727 Hz) | **F4**  (3591 Hz) |
| --- | --- | --- | --- | --- |
| **Ponsot et al., 2018** | 617 ± 83  t(9) = 2.35, p<.05 | 1187 ±31  t(9) = .4143, p>.1 | 2719 ± 28  t(9) = -.88, p>.1 | 3593 ± 22  t(9) = .35, p>.1 |
| **Present study** | 645 ± 71  t(22) = 6.09, p<.001 | 1193 ± 27  t(22) = 1.8, p<.1 | 2733 ± 38  t(22) =. 77, p>.1 | 3601 ± 26  t(22) = 1.9, p<.1 |
| **Comparison between Ponsot and Present study** | t(15) = -.96, p>.1 | t(15) = -.53, p>.1 | t(23) = -1.17, p>.1 | t(21) = -.91, p>.1 |

Table S1: Acoustic characteristics of the sounds before and after the application of the smiling Filter in both groups. Formants frequencies are presented in Hertz (mean ± sd), μ corresponds to the frequency value of the formant in the base sound. Results of the 1-sample t-test of the comparison between the value of the formants of the original sound and the modification of smile model.

# Motor resonance – effect of the muscle

Congruent responses to a smile could be associated with an increase in Zygomaticus major (ZM) activity compared to Corrugator Supercilii (CS) [10]. To test this assumption, a comparison between the muscular strength of CS and ZM was performed. A repeated-measures ANOVA was conducted to compare mean muscular activity in the 2900-3400 ms time window. A main effect of muscle was observed (F(1,23) = 15.70, p < .001, η² = .20), with significantly greater activity in CS (12.2 ± 10 a.u.) compared to ZM (-29.6 ± 6 a.u.) (Figure S2).


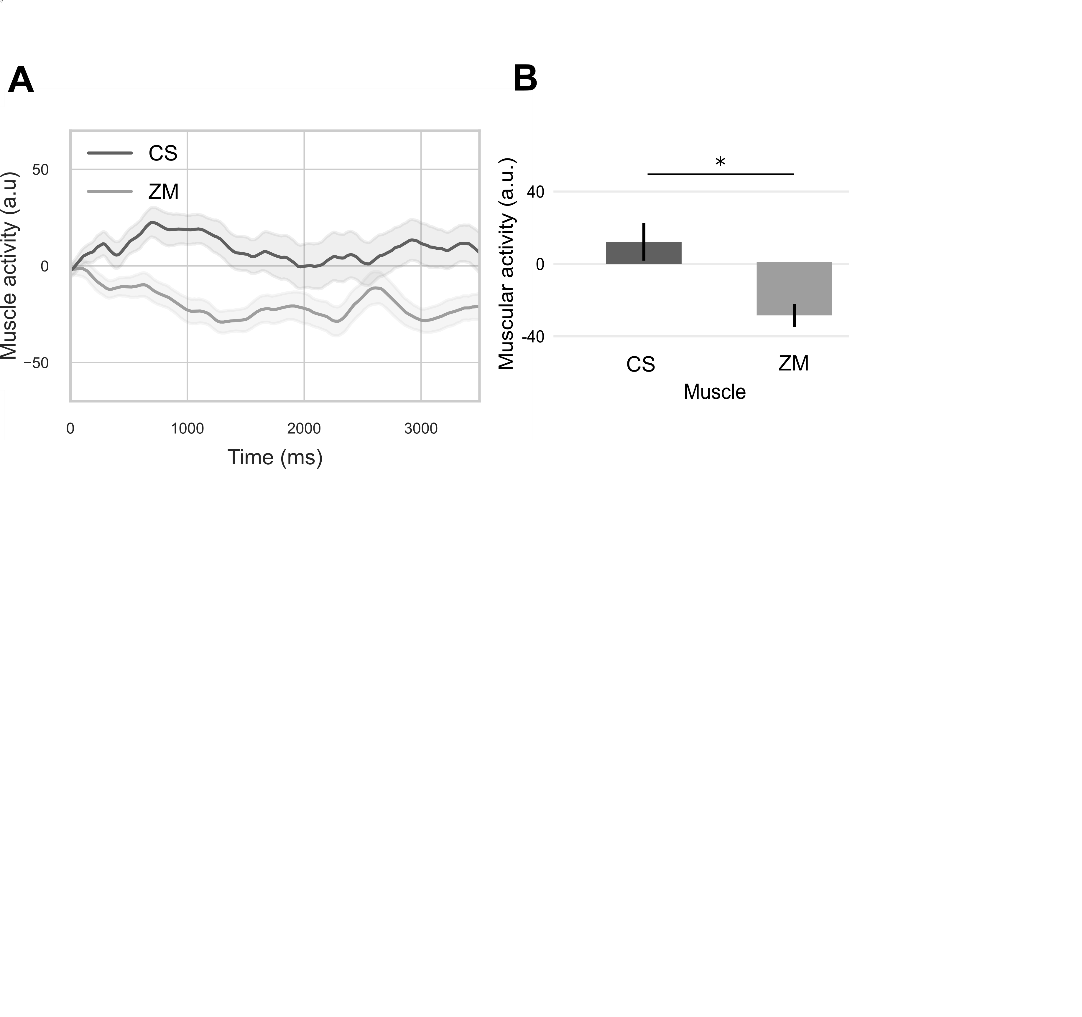


Figure S2: Zygomaticus major (ZM) and Corrugator supercilii (CS) activity. A: Effect of muscle; B: Effect of muscle (CS and ZM) mean response amplitude in the window 2900-3400ms.Shaded areas represent standard error of the mean. * p<.05

The activity of CS was more pronounced than the activity of ZM. CS activity was more discriminating than ZM activity, which can be explained by the different physiology of these two facial muscles. Despite a common innervation by the facial nerve, they do not depend on the same branch, with CS receiving more nerve impulses than ZM. Additionally, there is less muscular interference in CS than in ZM, making measurement easier and more accurate [11,12,13].

# Motor resonance – effect of Correct and Incorrect Response

To assess the effect of Response and its interaction with Choice, a repeated-measures ANOVA of muscle activity mean amplitude was performed, with post-hoc pairwise comparisons using Bonferroni correction to further specify significant interaction effects within Correct and Incorrect Responses.

The same time window (2900-3400 ms) was selected for both muscles, and a separate analysis was conducted. A repeated-measures ANOVA was performed to analyze the effects of Choice (Smile vs. Unsmile), Response (Correct vs. Incorrect), and the interaction between these two factors on mean muscular activity.

For ZM, no main effects were observed for either Response (F(1,23) = 0.28, p > .1, η² = 0.002) or Choice (F(1,23) = 0.66, p > .1, η² = 0.01). However, a significant interaction between these two factors was revealed (F(1,23) = 4.73, p < .05, η² = 0.06). To inspect relevant effects, comparisons within Correct and Incorrect responses were performed with a Bonferroni correction. This analysis revealed a tendency toward a difference between Correct responses according to Choice (t(23) = 2.24, p_corr < 0.1). (Figure S3Figure S3). No difference was observed between Incorrect responses (t(23) = -1.07, p_corr_> 0.1). The significant interaction between Choice and Response confirmed the Filter effect of the main analysis. However, post-hoc comparisons were not significant, which might be the consequence of the subdivision of Filter factor.


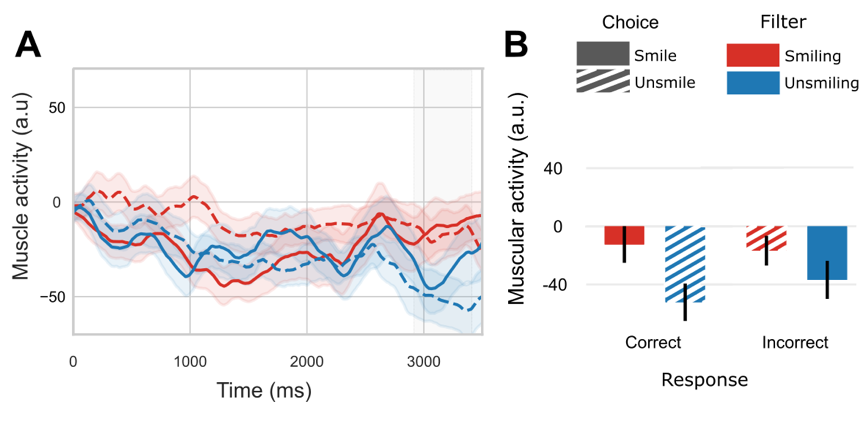


Figure S3: A: Effect of Filter, Choice and Response on ZM activity (without neutral sentences). Shaded areas represent standard error of the mean. B: Barplots represent mean pupil diameter in the window 2900-3400ms (mean ± standard error of the mean).

For CS, no main effect was observed for Response (F(1,23) = 3.33, p>.05, η^2^ = .01). A main effect of Choice was revealed (F(1,23) = 10.18 , p<.01, η^2^ = .09). No significant interaction between these two factors was demonstrated (F(1,23) = 1.89, p>.1, η^2^ = .01) (Figure S4). Main effect of choice was the same as demonstrated in the previous analysis, confirming that the expression of the emotional explicit motor resonance was supported by CS.


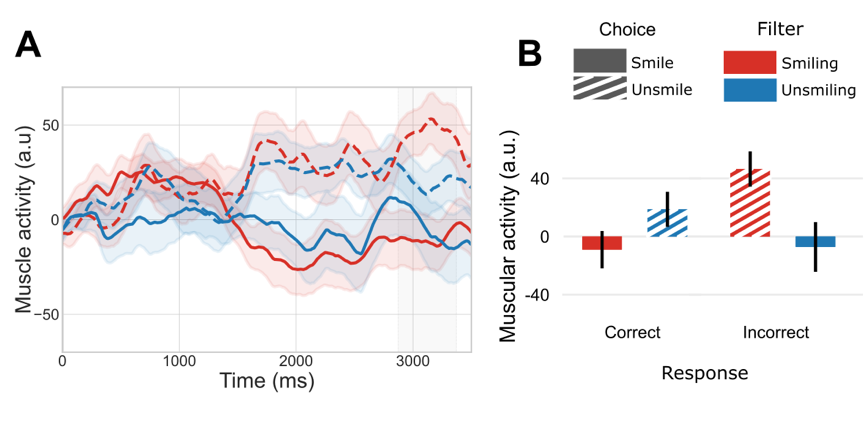


Figure S4: A: Effect of Filter, Choice and Response on CS activity (without neutral sentences). Shaded areas represent standard error of the mean. B: Barplots represent mean pupil diameter in the window 2900-3400ms (mean ± standard error of the mean).

# Correlations between physiological measurements

To find a potential link between physiological measures, rating, and EQ scores, a Pearson’s correlation matrix was calculated between the discriminating physiological variables (Filter effect on ZM and pupil activity, and Choice effect on CS activity) and accuracy. To highlight these links, for each measure, a difference between responses according to Filter or Choice was calculated, always making this difference in the direction of the effect (e.g., for the ZM, the difference between the response to the Smiling Filter and the response to the Unsmiling filter).


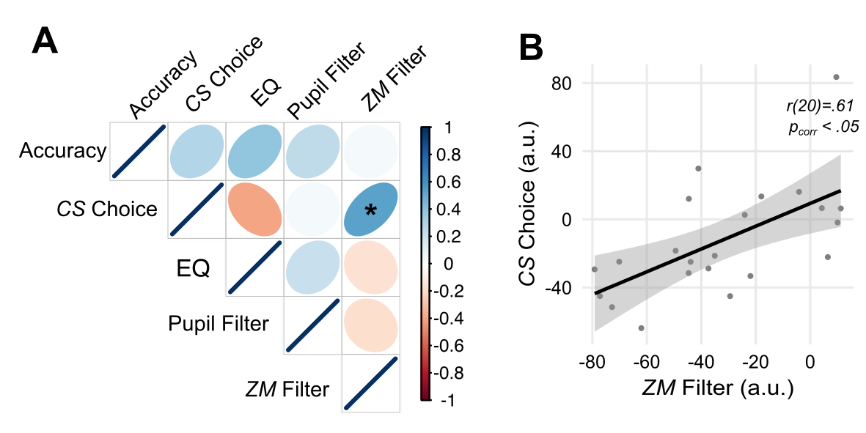


Figure S5 A: Pearson’s correlation matrix between physiological measures, rating responses and EQ scores with Bonferroni correction. The scale represents the value of the correlation coefficient (r). Accuracy: percentage of good rating of sentences (Smile and Unsmile). CS Choice: difference between the CS response to rated Unsmile and rated Smile sentences (2900-3400ms). Pupil Filter: difference between pupil diameter to Smiling and Unsmiling sentences (2100-2600ms). ZM Filter: difference between ZM response to Smiling and Unsmiling sentences (2900-3400ms). EQ: Empathic quotient; B: Correlation between CS activity according to given Choice and ZM activity according to Filter. * p_corr_<.05

No correlation was observed between the measures of fEMG and pupillometry, nor between EQ or Accuracy and any other parameter. However, the correlation between CS activity according to Choice and ZM activity according to Filter was significant (r(20) = .61, p_corr_< .05) (Figure S5A). The stronger CS reacted to Choice, the stronger ZM reacted to Filter (Figure S5B).

# References

[1] Russ, J. B., Gur, R. C., & Bilker, W. B. (2008). Validation of affective and neutral sentence content for prosodic testing. *Behavior Research Methods*, *40*(4), 935‑939. https://doi.org/10.3758/BRM.40.4.935

[2] Arias, P., Belin, P., & Aucouturier, J.-J. (2018). Auditory smiles trigger unconscious facial imitation. *Current Biology*, *28*(14), R782‑R783. https://doi.org/10.1016/j.cub.2018.05.084

[3] Arias, P., Bellmann, C., & Aucouturier, J.-J. (2021). Facial mimicry in the congenitally blind. *Current Biology*, *31*(19), R1112–R1114. https://doi.org/10.1016/j.cub.2021.08.059

[4] Ponsot, E., Arias, P., & Aucouturier, J.-J. (2018). Uncovering mental representations of smiled speech using reverse correlation. *The Journal of the Acoustical Society of America*, *143*(1), EL19‑EL24. https://doi.org/10.1121/1.5020989

[5] Burgess, A. E., & Colborne, B. (1988). Visual signal detection. IV. Observer inconsistency. *JOSA A*, *5*(4), 617‑627. https://doi.org/10.1364/JOSAA.5.000617

[6] Green, D. M. (1964). Consistency of auditory detection judgments. *Psychological Review*, *71*(5), 392‑407. https://doi.org/10.1037/h0044520

[7] Neri, P. (2010). How inherently noisy is human sensory processing? *Psychonomic Bulletin & Review*, *17*(6), 802‑808. https://doi.org/10.3758/PBR.17.6.802

[8] Burred, J. J., Ponsot, E., Goupil, L., Liuni, M., & Aucouturier, J.-J. (2019). CLEESE : An open-source audio-transformation toolbox for data-driven experiments in speech and music cognition. *PLOS ONE*, *14*(4), e0205943. https://doi.org/10.1371/journal.pone.0205943

[9] Boersma, P. (2002). Praat, a system for doing phonetics by computer. *Glot International*, *5*, 341‑345.

[10] Hess, U., Arslan, R., Mauersberger, H., Blaison, C., Dufner, M., Denissen, J. J. A., & Ziegler, M. (2017). Reliability of surface facial electromyography. *Psychophysiology*, *54*(1), 12‑23. https://doi.org/10.1111/psyp.12676

[11] Cacioppo, J. T., Tassinary, L. G., & Berntson, G. G. (2000). Psychophysiological science. *Handbook of Psychophysiology*, *2*, 3‑23.

[12] Ekman, P., & Friesen, W. V. (1975). *Unmasking the face englewood cliffs*. Spectrum-Prentice Hall.

[13] Rinn, W. E. (1984). The neuropsychology of facial expression : A review of the neurological and psychological mechanisms for producing facial expressions. *Psychological Bulletin*, *95*(1), 52‑77. https://doi.org/10.1037/0033-2909.95.1.52
